# The genotypic characterization of *Streptococcus pluranimalium* from aborted bovine fetuses in British Columbia, Canada

**DOI:** 10.3389/fmicb.2025.1603770

**Published:** 2025-06-12

**Authors:** Marcus Yee, Michael J. Trimble, Kazal Ghosh, Giselle Hughes, Daniel Knowles, Jun Duan, Stephen Raverty, Glenna McGregor, William W. L. Hsiao

**Affiliations:** ^1^Department of Molecular Biology and Biochemistry, Simon Fraser University, Burnaby, BC, Canada; ^2^Department of Health Sciences, Simon Fraser University, Burnaby, BC, Canada; ^3^Animal Health Centre, Ministry of Agriculture and Food, Government of British Columbia, Abbotsford, BC, Canada; ^4^Faculty of Health Sciences, Simon Fraser University, Burnaby, BC, Canada

**Keywords:** *Streptococcus pluranimalium*, bovine abortion, whole genome sequencing, antimicrobial resistance (AMR), animal health

## Abstract

**Introduction:**

Bovine abortions result in significant economic losses to dairy producers, and bacteria are among the most common causes of these abortions. In 2021, *Streptococcus pluranimalium* was isolated from a dairy abortion case for the first time in British Columbia (BC), Canada. This bacterium has previously been recovered from the reproductive tracts of dairy cattle and various other species, including humans.

**Methods:**

Between 2021 and 2023, *S. pluranimalium* was isolated from the placenta, fetal lung, and/or fetal abomasal contents of 10 aborted dairy fetuses submitted for routine abortion diagnostics. This study was conducted to better characterize the genotype of these 10 isolates. The histopathology of the bovine abortions was examined, and the BC strains were sequenced using Nanopore technology and underwent bioinformatic analysis.

**Results:**

The BC strains had an average genome size of 2,313,582 base pairs and an average GC content of 38.59%. Based on whole genome phylogeny, the BC strains were clustered together and distinctly separated from other publicly available strains of this species from different regions and isolation sources. Through Clusters of Orthologous Groups analysis, the BC strains contained a larger proportion of genes associated with the mobilome. Additionally, although we identified only a few antibiotic resistance genes or virulence factors (VFs) in these strains, several of these genes were located within prophage sequences.

**Discussion:**

Although the clinical and pathological significance of these bacteria in most abortion cases remains unclear, our findings underscore the importance of continued surveillance and research into uncommon pathogens to better understand their biology and potential impact on human and animal health.

## 1 Introduction

Bovine abortions result in significant economic losses for dairy producers worldwide. Reported bovine abortion rates range from 3 to 10% and can reach up to 30% in some herds. These abortions are attributed to a variety of factors, including genetic abnormalities, pathogen exposure, nutritional conditions, iatrogenic (medications) and teratogenic compounds, and hormonal fluctuations (Thurmond et al., 2005; Mee, 2023). Abortions are associated with various etiologic agents, including bacteria, fungi, protozoa, and viruses. Among these pathogens, infections caused by bacteria contribute significantly to morbidity, accounting for approximately 32–58% of all bovine abortion cases (Hecker et al., 2023). Pathogen exposure and invasion generally occur retrograde via the lower urogenital tract, or hematogenously through bacteremia, leading to localization in the placenta and developing fetus. These infections may be opportunistic, associated with normal commensal organisms, or result from exposure to contagious pathogens.

The dairy industry is the third largest agricultural sector in Canada (Pierre, 2017). In British Columbia (BC), particularly in the Fraser Valley, milk production plays a vital role in the regional economy. Due to the significant impact of fetal loss on dairy production, there has been an ongoing survey of bovine abortions at the Animal Health Centre (AHC) in Abbotsford, BC, to identify the causes of fetal loss. Between 2021 and 2023, a novel microbe, *Streptococcus pluranimalium,* was recovered from 10 aborted bovine fetuses. Prior to this period, this bacterium had not been identified in any bovine fetal samples submitted to the AHC. Given the limited knowledge about the natural history of this bacterium and the unknown risk of zoonotic transmission, a One Health approach was adopted to better characterize the molecular features of these isolates.

*S. pluranimalium* is a Gram-positive bacterium that is most closely related to *Streptococcus hyovaginalis*, *Streptococcus halotolerans*, and *Streptococcus thoraltensis* (Pan et al., 2018). The species has a broad tissue tropism and is capable of infecting a wide array of hosts, including canaries, chickens, cats, goats, and tilapia (Pan et al., 2018; Ghazvini et al., 2019). In bovine cases, *S. pluranimalium* has been linked to various diseases, such as vulvovaginitis, brain abscesses, tonsillitis, and mastitis, and reproductive issues, such as abortion (Foster et al., 2008; Twomey et al., 2012). Typically, this species transmits through blood, milk, and other infectious secretions from animals (Pan et al., 2018). There have been few reported cases of human infections, which manifested as suppurative meningitis, brain abscesses, endocarditis, and septicemia (Aryasinghe et al., 2014; Ananieva et al., 2023). Despite its wide range of potential hosts and zoonotic potential, there are few publicly available genomes of this species. As the genotype of this bacterium has not been well characterized, we performed whole-genome sequencing of 10 *S. pluranimalium* strains isolated from aborted bovine fetuses.

## 2 Materials and methods

### 2.1 Case material

The Animal Health Centre (AHC) is the provincial veterinary diagnostic laboratory for British Columbia and is accredited by the American Association of Veterinary Laboratory Diagnosticians (AAVLD). Case accessions included a range of tissue samples and whole carcasses obtained from local producers, veterinarians, and the general public. Bovine fetuses presented for necropsy underwent a standardized protocol, during which body weight and crown-to-rump length are recorded. All organ systems were examined internally and externally, and tissue samples were collected for routine bacteriology, histopathology, and other ancillary testing as deemed appropriate by a veterinary pathologist. Samples for bacteriology included the abomasal content, placenta, and lung tissues. These tissues were processed using conventional techniques, which included initial surface searing, followed by inoculation onto blood agar and MacConkey agar plates (Oxoid, ON). The samples were then incubated aerobically for up to 48 h. For any abortion cases, selective *Salmonella* culture was performed on Hektoen and XLT4 agar plates (Oxoid, ON), followed by selective enrichment in selenite broth. The colonies that grew on the agar plates were identified using biochemical tests and MALDI-TOF mass spectrometry (Bruker, ON), and the results were recorded in the proprietary veterinary information management system, VADDS&Vetstar developed by Advanced Technology Corp (Ramsey, NJ). For this study, *S. pluranimalium* isolates were archived at -20C for further analysis. The subsamples of the isolates were stored at -80C. Additional tissue samples were systematically collected for histopathological analysis, processed with an automated processor, embedded in paraffin, and sectioned at 5 *u*m. These sections were stained with hematoxylin and eosin using an automated stainer, cover-slipped, and then reviewed by board-certified veterinary pathologists. Microscopic lesions were graded on a scale from 0 (no apparent lesions) to 4 (severe lesions). A summary case report was prepared. Based on the histopathology results, ancillary diagnostic studies were conducted to screen for *Neospora caninum*, bovine viral diarrhea, *Chlamydophila abortus*, and *Ureoplasma diversum* (Hewinson et al., 1997), along with radial immunodiffusion testing for bovine IgG and IgM in select cases.

To determine the number of *S. pluranimalium* isolates recovered at the AHC, a database search covering the period from 2008 to 2024 was performed using the parameters of bovine, fetus, and abortion.

### 2.2 Sample extraction, library preparation, and sequencing

Bacterial isolates were recovered from freezer stocks by initial culturing on tryptic soy agar plates supplemented with 5% defibrinated sheep’s blood and incubating overnight at 37°C. A single colony was picked and inoculated into tryptic soy broth supplemented with 5% defibrinated sheep’s blood, and the culture was shaken overnight at 37°C. DNA extractions were performed using Qiagen’s DNeasy Blood & Tissue kit (Toronto, ON), following the supplemental steps for the pretreatment of Gram-positive bacteria. The extracted DNA was then assessed for purity and concentration using gel electrophoresis, Nanodrop, and Qubit.

The samples were then prepared for sequencing on MinION, Oxford Nanopore Technologies (**ONT**) (Oxford, UK), using their Native Barcoding Kit (EXP-NBD114). This process involved repairing the DNA and performing end-preparation using the NEBNext FFPE DNA Repair Mix and the NEBNext Ultra II End Repair/dA-Tailing Module reagents (New England Biolabs, NEB; Whitby, ON). The samples were purified using AMPure XP beads, and the native barcodes were then ligated onto the fragments using Blunt/TA Ligase Master Mix (NEB). The samples were purified using AMPure XP beads, quantified, and then pooled in equimolar amounts. The ONT adapters were ligated to the barcoded pool using T4 Ligase (NEB) before being loaded onto an R10.3 flow cell, following ONT specifications. The samples were sequenced for 72 h and base-called using the MinKNOW (v22.03.6) high-accuracy base-calling model.

### 2.3 Sequence analysis

Raw reads had adaptors trimmed using Porechop v0.2.4 (Wick, 2018) and were quality-filtered using NanoFilt v2.8.0 (using the parameters -q 10, −l 300, −-headcrop 40) (De Coster et al., 2018). The filtered reads were assembled using Flye v2.9.1 (Kolmogorov et al., 2019) and polished using Medaka v1.11.1 (ONT, 2024). The genomes were annotated using Bakta v1.7 (Schwengers et al., 2021), and a Maximum likelihood phylogenetic tree was created using the tool autoMLST (accessed August 23, 2022) (Alanjary et al., 2019). Genome completeness was assessed using BUSCO v5.7.1 (Simão et al., 2015). The Virulence Factor Database (accessed May 10, 2024) (Liu et al., 2022) and Abricate v1.0.0 (Seemann, 2020) were used to search for the presence of virulence factors. The Comprehensive Antibiotic Resistance Database (CARD) v3.2.9 (McArthur et al., 2013), coupled with RGI v6.0.2, was used to examine antibiotic resistance genes (ARGs). Clusters of Orthologous Groups analysis was conducted using the EggNOG-mapper v2.1.9 (Cantalapiedra et al., 2021) and EggNOG Database v5.0.2 (Huerta-Cepas et al., 2018). Plasmids were identified using Mobsuite v3.0.3 (Robertson and Nash, 2018), and phage sequences were identified using Virsorter v2.2.3 (Guo et al., 2021).

## 3 Results

### 3.1 Identification of microorganisms in aborted fetuses

Between 2008 and 2024, 382 fetuses were submitted to the AHC for diagnostic evaluation. Each case was reviewed individually, and the signalment, gross pathology, histopathology, bacteriology, and molecular results were tabulated and scored. Using MALDI-TOF mass spectrometry, *S. pluranimalium* was identified in 10 fetal samples, which then underwent further characterization. In these 10 samples, additional bacterial isolates were taxonomically identified at the species level or, if the species level could not be determined, at the genus level. Overall, 13 bacterial species and genera were isolated from the lung, stomach contents, and placenta (Supplementary Table 1). These included *S. pluranimalium* (*n* = 11), *Streptococcus uberis* (*n* = 2), the *Glutamicibacter* genus (*n* = 2), the *Staphylococcus* genus (*n* = 3), *Enterococcus saccharolyticus* (*n* = 1), the *Psychrobacter* genus (*n* = 1), the *Arthrobacter* genus (*n* = 3), *Aerococcus viridans* (*n* = 1), the *Vibrio* genus (*n* = 1), the *Acinetobacter genus* (*n* = 5), the *Corynebacterium* genus (*n* = 2), *Enterococcus faecium* (*n* = 1), and *Escherichia coli* (*n* = 5). In addition, sample BC-AHC-08 contained the fungi *Aspergillus fumigatus* and a species of *Candida*. The presence of three other pathogens—*Neospora caninum* (*n* = 8), Bovine Viral Diarrhea Virus (*n* = 1), and *Ureoplasma diversum* (*n* = 1)—was confirmed either through polymerase chain reaction or serology.

### 3.2 Lesion distribution

The histopathology results indicated the presence of several lesions in the aborted fetuses (Table 1), affecting various tissues including the brain, lungs, heart, muscles, kidneys, and eyes. Lesions in the eyes (*n* = 6) and placenta (*n* = 7) were the most common. Sample BC-AHC-03 exhibited the most extensive lesions, affecting six different tissues. No histopathological abnormalities were apparent in sample BC-AHC-05.

**Table 1:** Histopathological findings of aborted bovine fetuses.

Case ID	Myositis	Myocarditis	Meningoencephalitis	Placentitis	Nephritis	Pneumonia	Cholangiohepatitis	Palpebritis/conjunctivitis
BC-AHC-01	0	0	0	2	0	0	N/A	N/A
BC-AHC-02	2	4	2	2	2	0	N/A	N/A
BC-AHC-03	1	1	0	3	1	0	2	1
BC-AHC-04	0	1	0	1	1	0	1	1
BC-AHC-05	N/A	N/A	N/A	N/A	N/A	N/A	N/A	N/A
BC-AHC-06	N/A	4	N/A	1	1	0	1	1
BC-AHC-07	0	0	0	0	0	0	1	0
BC-AHC-08	0	0	0	4	0	2	0	2
BC-AHC-09	1	0	N/A	N/A	0	1	0	1

Lesions were assessed across various tissue types and were scored based on severity on a scale from 0 (no apparent lesions) to 4 (severe lesions). It is important to note that mixed bacterial or bacterial and fungal or protozoal infections likely contributed to the observed microscopic lesions.

### 3.3 Whole-genome sequencing of *Streptococcus pluranimalium*

Among the 10 fetal samples with MALDI-TOF-confirmed *S. pluranimalium*, isolates from 9 were further characterized using molecular methods. Whole-genome sequencing was performed on one isolate from each sample, except for sample BC-AHC-09, from which two isolates were sequenced: BC-AHC-09_a isolated from the lung and BC-AHC-09_b isolated from the stomach. The BC strains had an average genome size of 2,313,582 base pairs (bp) and an average GC content of 38.59%, similar to the reference strain TH11417 (Pan et al., 2018), which has a genome size of 2,065,522 and a GC content of 38.65 (Table 2). The BC strains had an average of 15.2 contigs per assembly and an average genome completeness of 96.63%, as assigned by BUSCO.

**Table 2:** Whole-genome sequence assembly statistics of the BC *S. pluranimalium* strains.

Strain ID	No. of contigs	Genome size (Mb)	GC (%)	N50 (Mb)	Genome completeness (%)
BC-AHC-01_a	12	2.4	38.54	1.6	98.0
BC-AHC-02_a	5	2.2	38.59	2.2	99.0
BC-AHC-03_a	13	2.1	38.68	2.1	98.0
BC-AHC-04_a	22	2.5	38.87	1.5	91.5
BC-AHC-05_a	17	2.3	38.5	2.3	95.0
BC-AHC-06_a	10	2	38.45	2	97.5
BC-AHC-07_a	31	2.4	38.59	2.3	97.7
BC-AHC-08_a	18	2.4	38.5	0.9	91.5
BC-AHC-09_a	14	2.3	38.66	2.2	90.0
BC-AHC-09_b	19	2.4	38.56	2.3	91.5
Reference TH11417 (Complete Genome)	1	2.1	38.65	2.1	98.8

### 3.4 Phylogeny

**Figure 1:**
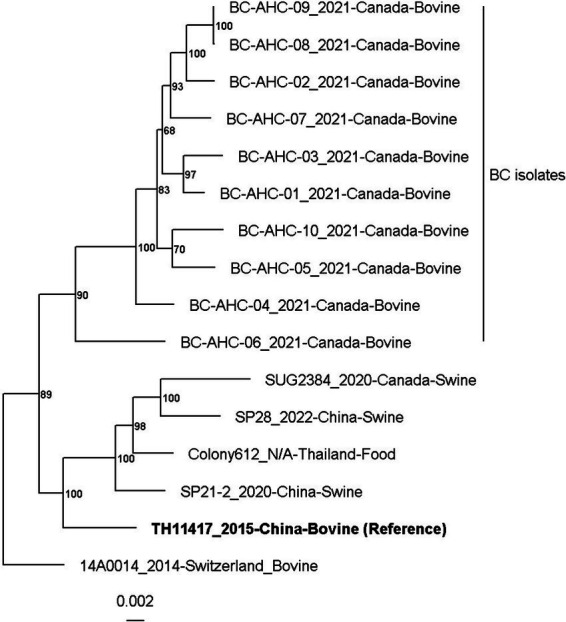
Maximum likelihood phylogenetic tree of *S. pluranimalium* strains with multilocus sequence typing analysis using the tool autoMLST.

A maximum likelihood phylogenetic tree was constructed based on the analysis of core, single-copy genes present in all strains (Figure 1). The phylogenetic tree was constructed using the 10 BC genomes and 6 other publicly available genomes from the NCBI as of July 2024. The six genomes from the NCBI include TH11417 (Pan et al., 2018), 14A0014 (Rodriguez Campos et al., 2018), Colony612 (Buathong et al., 2021), SP21-2 (Xu et al., 2023), SP28 (Zhu, 2023), and SUG2384 (Holman et al., 2024). The BC strains formed a distinct clade, with strain BC-AHC-06_a being the most divergent. Strains BC-AHC-09_a and BC-AHC-09_b were isolated from the same sample but were separated on the phylogenetic tree. The publicly available strain, 14A0014, which was also recovered from a bovine abortion sample, was the most genetically distinct compared to the BC strains.

### 3.5 Clusters of orthologous groups

**Figure 2:**
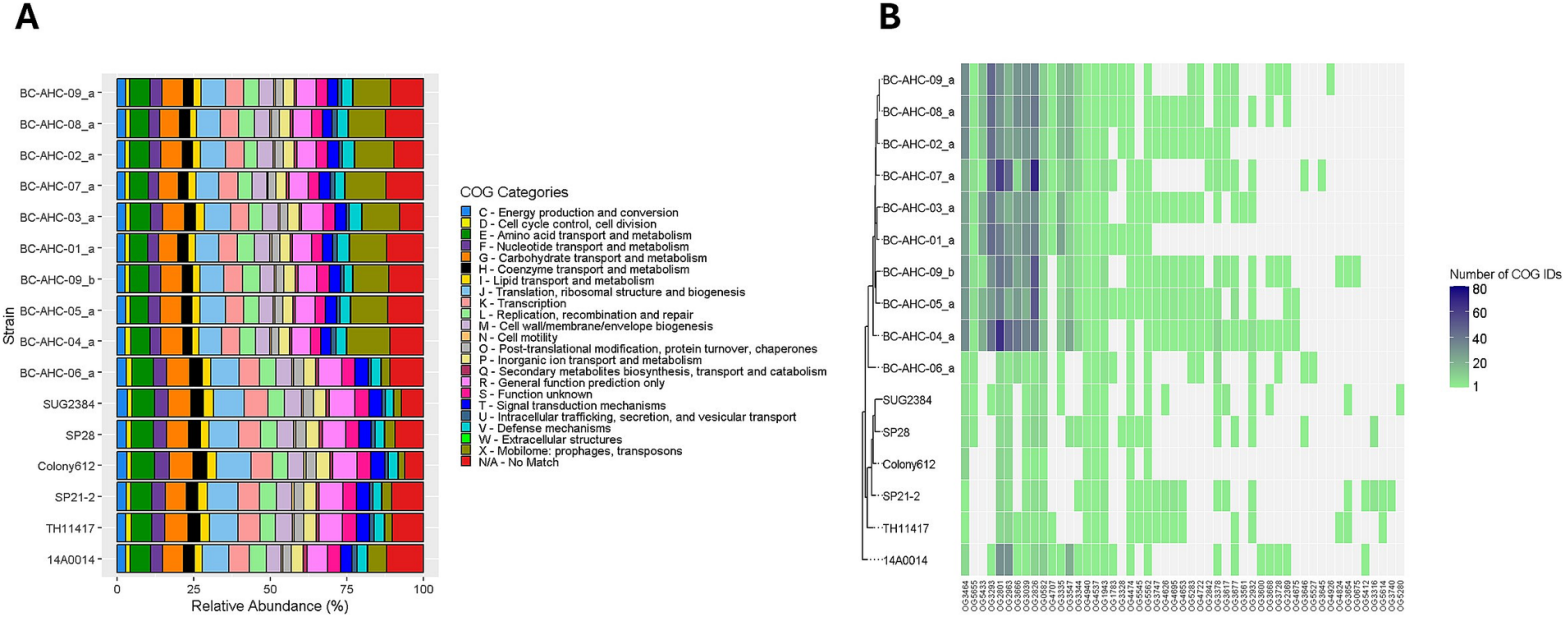
**(A)** Distribution of COG functional categories of each gene in *S. pluranimalium* genomes. **(B)** Heatmap showing the number of COG IDs associated with Category X in the *S. pluranimalium* genomes. COG IDs not present in a strain are colored light gray, while COG IDs present in a strain are represented by a gradient from light green to navy blue.

Clusters of orthologous groups (COGs) is a method of phylogenetic classification that clusters each gene into a COG ID and assigns each COG ID to a functional category. The COG analysis, supported by Fisher’s exact test, revealed that the BC strains, except for strain BC-AHC-06_a, contained a significantly higher proportion of COG IDs associated with category X compared to *other S. pluranimalium* strains (*p* < 0.05) (Figure 2A). The BC strains, excluding BC-AHC-06_a, had an average of 12.5% of all COG IDs associated with category X. In comparison, strain BC-AHC-06_a had only 2.7% of its COG IDs associated with category X, which is similar to the non-BC strains that had an average of 3.2% of their COG IDs. With the exception of strain BC-AHC-06_a, many COG IDs associated with category X that were in high abundance were shared among all of the BC strains (Figure 2B).

### 3.6 Virulence factors

Utilizing the tool Abricate and the Virulence Factor Database, seven different virulence factors (VFs) were identified in the BC strains, encompassing four virulence classes: immune modulation, stress survival, exotoxin, and adherence (Table 3). All BC strains contained the gene cluster *rfbA*, *rfbB*, and *rfbC*, with an 80–82% nucleotide identity. The VFs *tig/ropA*, *plr/gapA*, and *eno* were also found in all BC strains, with identities of approximately 82, 89, and 90%, respectively. Strain BC-AHC-06_a contained the *SSU98_0978* virulence factor, with a nucleotide identity of 95%.

**Table 3:** Percent identity of virulence factors in *S. pluranimalium* strains, identified using the Virulence Factor Database.

Strains	rfbA	rfbB	rfbC	tig/ropA	plr/gapA	eno	SSU98_0978	SAG_RS09105
BC-AHC-01_a	80.6	81.06	81.82	82.4	88.97	89.82		
BC-AHC-02_a	80.6	81.06	81.82	82.24	88.27	90.05		
BC-AHC-03_a	80.6	80.87	81.82	82.4	89.26	89.82		
BC-AHC-04_a	80.48	81.06	81.48	82.4	89.17	90.13	95.46	
BC-AHC-05_a	80.48	80.79	81.48	82.24	89.07	89.9		
BC-AHC-06_a	81.06	81.16	81.48	82.24	88.77	90.13		
BC-AHC-07_a	80.6	80.98	81.48	82.17	88.27	89.9		
BC-AHC-08_a	80.71	81.06	81.48	82.24	88.27	89.9		
BC-AHC-09_a	80.71	81.06	81.48	82.24	88.27	89.9		
BC-AHC-09_b	80.48	81.16	81.82	82.17	88.97	89.9		
TH11417	80.37	81.16	81.82	82.24	89.26	90.05		
A40014	80.94	81.27	80.64	82.56	89.26	90.21		95.9
Colony612	80.71	81.06	81.45	82.4	88.87	90.21		95.86
SP-21-2	80.71	81.16	81.48	82.4	89.36	90.21		94.11
SP28	80.71	81.26	81.48	82.4	88.87	90.28		95.9
SUG2384	81.17	81.16	81.48	82.17	88.57	90.21		

A threshold of 80% identity and 80% coverage relative to a reference virulence factor was applied.

### 3.7 Antimicrobial resistance genes

**Figure 3:**
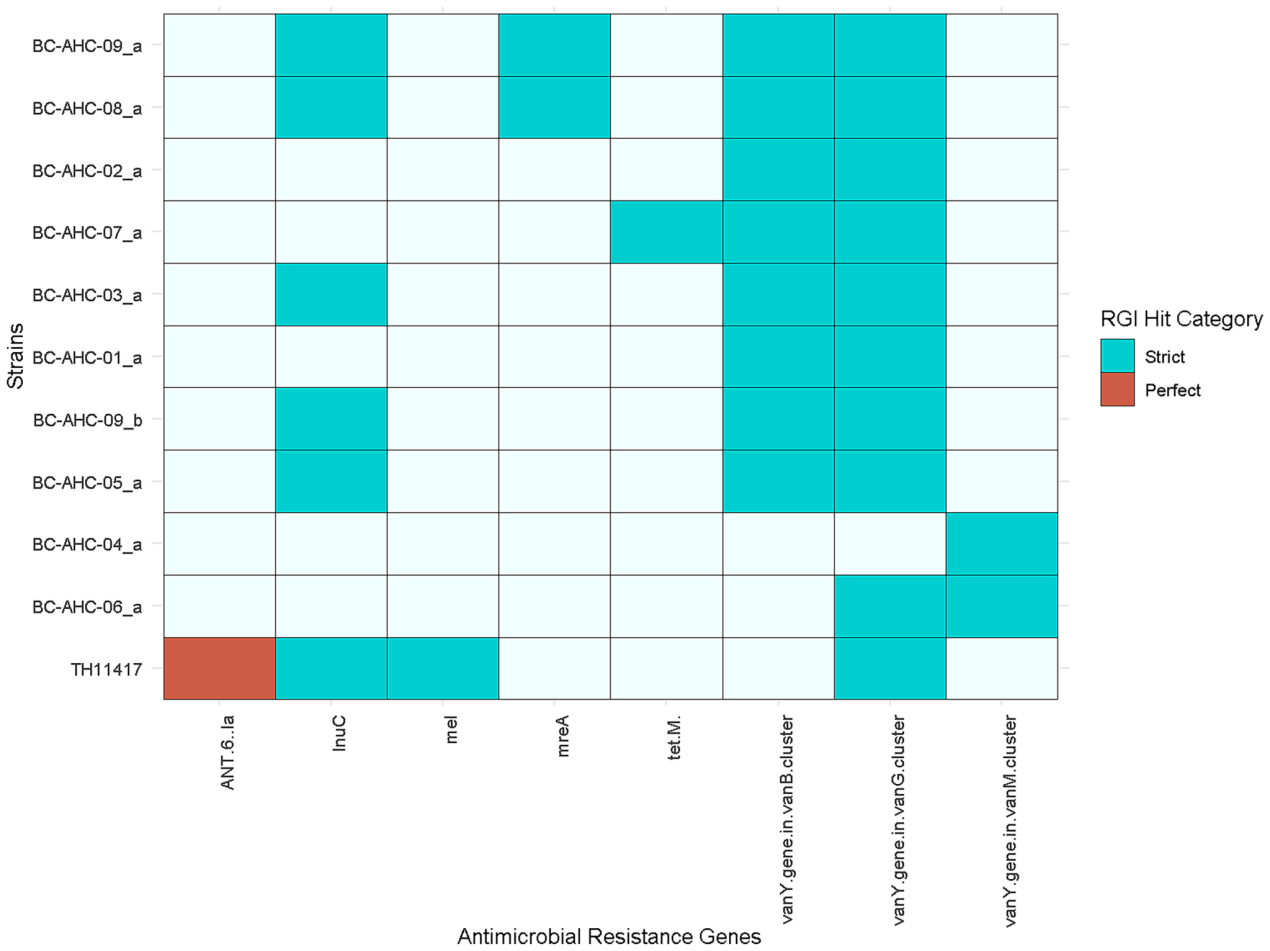
Presence of ARGs identified using the Resistance Gene identifier tool and the Comprehensive Antibiotic Resistance Gene Database. Perfect and strict hits were used.

Utilizing the Resistance Gene Identifier tool and the Comprehensive Antibiotic Resistance Database, several genes conferring resistance to multiple antibiotics were identified using strict and perfect criteria (Figure 3). Antibiotic resistance genes (ARGs) conferring resistance to a number of antibiotics such as glycopeptides (*vanY* genes), lincosamides (*lnuC*), tetracycline (*tet(M)*), and macrolides (*mreA*) were detected (Table 2). Strains BC-AHC-08_a and BC-AHC-09_a contained the largest number of ARGs, with four each.

### 3.8 Plasmids

Using the tool MOB-suite, two novel plasmids were identified. The first was a 10.8 kb plasmid identified in strains BC-AHC-01_a and BC-AHC-02_a, and the second was a 5.4 kb plasmid identified in strains BC-AHC-03_a, BC-AHC-04_a, BC-AHC-08_a, and BC-AHC-09_b. Both plasmids showed the closest similarity to NSUI060 (Athey et al., 2016), a 5.6 kb *Streptococcus suis* plasmid. These *S. pluranimalium* plasmids did not contain any known virulence factors or antimicrobial resistance genes, had the MOBV relaxase type, and were classified as mobilizable.

### 3.9 Prophages

Using VirSorter2, a machine learning tool that predicts phage sequences based on features such as the number of hallmark viral genes, the percentage of viral genes, and the mean GC content, we identified 41 putative prophages in the BC strains (Supplementary Table 2). Their lengths ranged from 1,024 bp to 295,435 bp. Each BC strain had an average of 291,212 bp of phage sequences. In comparison, the non-BC strains Colony612 and SP28 did not contain any phage sequences. The four other non-BC strains had an average of 147,501 bp of phage sequences per strain. Strains BC-AHC-03_a and BC-AHC-09_b contained two virulence factors, tig/ropA and plr/gapA, located on a phage sequence. The *eno* virulence factor was also located on a phage sequence in strain BC-AHC-06_a. Strain BC-AHC-08_a contained a phage sequence with three ARGs: the *vanY* gene in the *vanB* cluster, *mreA*, and *lnuC*. In addition, strain BC-AHC-09_a contained one phage sequence with one ARG, *lnuC*.

## 4 Discussion

Bovine abortions cause significant economic losses and are frequently associated with bacterial infections. Since 2021, *S. pluranimalium*, an emerging pathogen, has been identified in a subset of aborted fetuses in BC. Notably, MALDI-TOF analysis was only implemented at the AHC in 2018; prior to this period, the biochemical tests used for bacterial identification were not capable of distinguishing this pathogen. In addition to *S. pluranimalium*, 12 other bacterial isolates were recovered from these aborted fetuses. The most common bacterial species identified were *Escherichia coli* (*n* = 5) and the *Acinetobacter* genus (*n* = 5). Evidence of exposure to the protozoan parasite, *Neospora caninum,* was also found in eight fetuses. Given the non-sterile conditions at the farm site, it is not unusual to observe a diversity of microbial isolates in the submitted case materials (Hecker et al., 2023). This situation complicates the determination of the cause of fetal death in cases of mixed infection bovine abortion (Barkallah et al., 2014). Although we cannot definitively state that *S. pluranimalium* was the cause of abortion, the discovery of this bacterium from multiple tissues with occasional active inflammation warrants further investigation.

In this study, we conducted whole-genome sequencing on 10 *S. pluranimalium* isolates, greatly increasing the number of publicly available genomes for this species (Table 2 and Supplementary Table 1). The phylogenetic analysis revealed a distinct BC clade, with strain BC-AHC-06_a being the most divergent. Strains BC-AHC-09_a and BC-AHC-09_b were both isolated from the same case, with one isolate recovered from the lung and the other from the stomach (Figure 1). These strains appeared separately on the phylogenetic tree, indicating a mixed infection of two *S. pluranimalium* strains. Interestingly, the public strain 14AA0014, which was also isolated in Canada and associated with bovine abortions, was the most distinct compared to BC strains.

In BC strains, we found a larger number of COG IDs linked to COG category X compared to the other *S. pluranimalium* strains (Figure 2B). This category is listed as the mobilome and encompasses genes associated with transposons, prophages, and plasmid replication (Galperin et al., 2014). The exception was strain BC-AHC-06_a, which was the most divergent strain on the phylogenetic tree. Analysis of the location of these COG X IDs showed that they were distributed evenly across the genomes. Although the BC strains contained more prophage sequences, as predicted by VirSorter2, and these phage sequences contained many COG category X genes, removing the phage-located COG IDs did not alter the overall trend. The BC strains, except for BC-AHC-06_a, still exhibited a significantly higher percentage of COG category X genes. Although these findings further substantiate the BC clade, differentiating the BC strains from other *S. pluranimalium* strains, further research is needed to understand the higher prevalence of genes associated with mobilizable elements in the BC strains.

Similar to other *S. pluranimalium* strains, the BC strains contained limited antimicrobial resistance genes. Several of the BC strains shared the *vanY* gene from the *vanG cluster* and the *lnuC* gene with the reference *S. pluranimalium* strain TH11417 (Figure 3) (Pan et al., 2018). The *vanY* gene confers resistance to glycopeptides, while the *lnuC* gene confers resistance to lincosamides. The BC-AHC-07_a strain contained the *tet(M)* gene, which confers resistance to tetracycline. This gene was not present in any other *S. pluranimalium* genome as of July 2024. Both BC and non-BC *S. pluranimalium* strains contained six virulence factors, indicating that these factors are likely part of the core genome for the species. These virulence genes include the gene cluster *rfbA, rfbB,* and *rfbC*. These genes are involved in capsule synthesis and are traditionally associated with O-antigen production in Gram-negative bacteria; however, they have been reported in *Streptococcus thermophilus* (Bank et al., 2022). The three other virulence factors present in all *S. pluranimalium* strains are *tig/ropA*, a trigger factor linked to stress tolerance, *plr/gapA*, a GAPDH homolog involved in host cell adherence, and *eno*, an enzyme linked to the binding of human plasminogen (Antikainen et al., 2007; Purves et al., 2010; Wu et al., 2011). BC-AHC-04_a was the only BC strain to have the virulence factor SSU98_0978, an agglutinin receptor involved in adhesion (Forsgren et al., 2010).

We also identified two novel plasmids, both showing the closest similarity to a *Streptococcus suis* plasmid from Canada (Athey et al., 2016). The larger plasmid, measuring 10.8 kb, was found in strains BC-AHC-01_a and BC-AHC-02_a. The smaller plasmid, measuring 5.4 kb, was found in strains BC-AHC-03_a, BC-AHC-04_a, BC-AHC-09_a, and BC-AHC-09_b. Interestingly, further analysis revealed that the larger plasmid in BC-AHC-01_a and BC-AHC-02_a appeared to be an expanded version of the smaller plasmid, representing a duplication of its genetic content. No known virulence factors or antimicrobial resistance genes were found on these plasmids.

The BC strains contained a higher number of longer phage sequences compared to the other *S. pluranimalium* strains. Notably, two virulence factors, *tig/ropA* and *plr/gapA,* were identified within a phage sequence in two BC strains. In addition, another BC strain contained a phage sequence with the *eno* virulence factor. One BC strain contained a phage sequence with three ARGs: *vanY* in the *vanB* cluster*, mreA*, and *lnuC*. In contrast, another strain contained a phage sequence with *lnuC*. As these virulence factors and ARGs present on the phage sequences are potentially mobilizable, these findings highlight the need for vigilant monitoring to prevent the potential transmission of these genes from *S. pluranimalium* to other species, given its broad host tropism.

## 5 Conclusion

We performed whole-genome sequencing of 10 BC *S. pluranimalium* isolates, a previously unreported species in this region. The BC strains clustered separately from other *S. pluranimalium* strains but shared similar attributes, including a low number of detected VFs and ARGs. Overall, our findings underscore the importance of continued surveillance and research on rare and novel bacterial isolates to enhance our understanding of their biology and potential impact on human and animal health.

## Data Availability

All genome sequences generated in this study have been deposited in the GenBank database under the BioProject accession number PRJNA1251123.

## Supplementary material

SUPPLEMENTARY TABLE 1 Sample metadata and Pathology.

SUPPLEMENTARY TABLE 2 Bacteriophages identified in *Streptococcus pluranimalium* strains.
